# Linear Cyclodextrin Polymer Prodrugs as Novel Therapeutics for Niemann-Pick Type C1 Disorder

**DOI:** 10.1038/s41598-018-27926-9

**Published:** 2018-06-22

**Authors:** Aditya Kulkarni, Paola Caporali, Atul Dolas, Soniya Johny, Sandeep Goyal, Jessica Dragotto, Alberto Macone, Ramesh Jayaraman, Maria Teresa Fiorenza

**Affiliations:** 1 Aten Porus Lifesciences, Bangalore, 560068 India; 2 Department of Psychology, Division of Neuroscience, Sapienza University, Rome, Italy; 3 Department of Biochemical Sciences “A. Rossi Fanelli”, Sapienza University of Rome, Rome, Italy; 4 TheraIndx Lifesciences Pvt Ltd., Bangalore, 562123 India; 5 IRCCS Fondazione Santa Lucia, Via del Fosso Fiorano 64, 00179 Rome, Italy

## Abstract

Niemann-Pick Type C1 disorder (NPC) is a rare lysosomal storage disease characterized by the accumulation of cholesterol in lysosomes. NPC has no FDA approved treatments yet, however 2-hydroxypropyl-β-cyclodextrin (HPβCD) has shown efficacy for treating the disease in both mouse and feline NPC models and is currently being investigated in late stage clinical trials. Despite promising results, therapeutic use of HPβCD is limited by the need for high doses, ototoxicity and intrathecal administration. These limitations can be attributed to its poor pharmacokinetic profile. In the attempt to overcome these limitations, we have designed a β-cyclodextrin (βCD) based polymer prodrugs (ORX-301) for an enhanced pharmacokinetic and biodistribution profile, which in turn can potentially provide an improved efficacy at lower doses. We demonstrated that subcutaneously injected ORX-301 extended the mean lifespan of NPC mice at a dosage 5-fold lower (800 mg/kg, body weight) the HPβCD dose proven efficacious (4000 mg/kg). We also show that ORX-301 penetrates the blood brain barrier and counteracts neurological impairment. These properties represent a substantial improvement and appear to overcome major limitations of presently available βCD-based therapy, demonstrating that this novel prodrug is a valuable alternative/complement for existing therapies.

## Introduction

Niemann-Pick Type C (NPC) disease is a rare lysosomal storage disorder affecting approximately 1:120,000 children globally^1^. The disease is caused by a mutation in either the NPC1 (95%) or NPC2 (5%) genes both of which are responsible for the transfer of unesterifed cholesterol (UC) from the late endosome/lysosome compartment (LE/LY) to the cytosol. A mutation in either of these genes causes the aberrant accumulation of cholesterol and other lipids, resulting in enlargement of the LE/LY compartment^2–4^. The clinical manifestations range from neurological symptoms such as ataxia, cognitive loss, seizures and dementia, to systemic defects as the enlargement of the liver and spleen^5,6^.

There are currently no Food and Drug Administration (FDA) approved treatments for NPC patients. Miglustat, a substrate reduction drug used for the treatment of Gaucher’s disease, has been approved for NPC in Europe, but not approved in the US^7–9^. Other potential therapeutics being investigated are Arimoclomol, a heat shock protein activator (Hsp70)^10^, and Vorinostat, a histone deacetylases (HDAC) inhibitor that has been approved by FDA for the treatment of T-cell lymphoma^11,12^. 2-Hydroxypropyl-β-cyclodextrin (HPβCD) is a FDA approved hydrophilic excipient that is known to bind to and solubilize cholesterol^13^. HPβCD has shown excellent efficacy towards mobilization of cholesterol in NPC cells^14^ and animal models^15–18^. Preclinical studies in NPC mouse models have demonstrated that a single intraperitoneal injection of HPβCD early in life significantly improves the lifespan and delays neurodegeneration^15^. Intrathecal administration of HPβCD also slowed the progression of neurological damage and improved survival of NPC mouse and feline models^19–22^. Intrathecal administration of the drug in a single NPC1 patient led to an increase in cholesterol redistribution in the central nervous system indicating similar efficacy^23^. In a recently concluded Phase 1/2 clinical trial of 14 patients, it was seen that intrathecal HPβCD slowed disease progression with an acceptable safety profile^24^. The drug is now undergoing Phase 2b/3 clinical trials and FDA has granted orphan drug designation to HPβCD for the treatment of NPC. Despite the promising results, HPβCD has several shortcomings for the treatment of NPC. Firstly, an extremely high concentration of the drug is required to clear cholesterol and elicit a therapeutic response. Secondly, the drug needs to be administered intrathecally to address the neurological symptoms since only about 0.2–1.5% reaches the brain upon systemic administration due to its inability to cross the blood brain barrier (BBB)^25,53^. Thirdly, it was seen in NPC mouse and feline models that the high doses of the drug cause high-frequency hearing loss^26,27^. The ototoxic effect of the drug was also observed in the Phase 1/2 clinical trial, where all patients suffered from additional hearing impairment that was attributed to outer hair cell loss^24^. Most of the issues faced by HPβCD can be largely attributed to the poor pharmacokinetic profile and bioavailability, which results in most of the drug being excreted through renal filtration^28,29^. This limits the effectiveness of the drug when administered systemically hence resulting in the need for high doses and intrathecal administration.

To address these issues, we designed a linear degradable high molecular weight polymer prodrug version of βCD, hereafter named ORX-301. The high molecular weight polymer was designed to have an improved pharmacokinetic and slower elimination profile resulting in an enhanced bioavailability and thus potentially higher efficacy at lower doses. While there have been previous attempts at developing drug delivery systems for βCD and its variants for NPC^30–36^, to our knowledge this is the first report demonstrating the efficacy of a cyclodextrin-based polymer prodrug in significantly improving survival and neurobehavioral performance in an animal model of NPC. In ORX-301, individual βCD moieties are linked together via a degradable ketal linkage. This strategy allowed for a high % CD loading of ~95% by weight of the polymer compared to previous attempts where the % loading was between 30–40%^30,31,36^. The ketal linkage allows for degradation of the polymer into low molecular weight excretable compounds at the acidic pH of lysosomes. Importantly, these low molecular weight degradation products of the polymer are βCD and acetone, both of which should be cleared easily and not cause bioaccumulation or toxicity. For instance, the released acetone should be metabolized to pyruvate and used for ATP production whereas the monomeric CD should undergo renal elimination^28,29,37,38^.

To validate our hypothesis we synthesized ORX-301 of MW 33,000 Da that was used for the proof-of-concept animal studies. We evaluated ORX-301 pharmacokinetic profile and bioavailability in healthy mice, along with its *in vivo* efficacy in NPC mice. We found that ORX-301 has an improved terminal half-life and bioavailability over HPβCD, and a MTD of 2000 mg/kg. Most importantly our *in vivo* efficacy experiments demonstrated that subcutaneously administered ORX-301 (800 mg/kg body weight) increased the mean lifespan of NPC mice from 110 ± 2.19 days to 170 ± 2.76 days. This improvement in survival is comparable to that observed when mice are treated with HPβCD at 4000 mg/kg, nearly 5 times the dose of ORX-301^34^, while NPC mice treated with HPβCD at 800 mg/kg had a mean lifespan of 150 ± 2.55 days. All together our results strongly suggest that ORX-301 can be a valuable therapeutic for NPC.

## Results

### ORX-301 polymer displays enhanced pharmacokinetics and bioavailability compared to HPβCD

**Figure 1:**
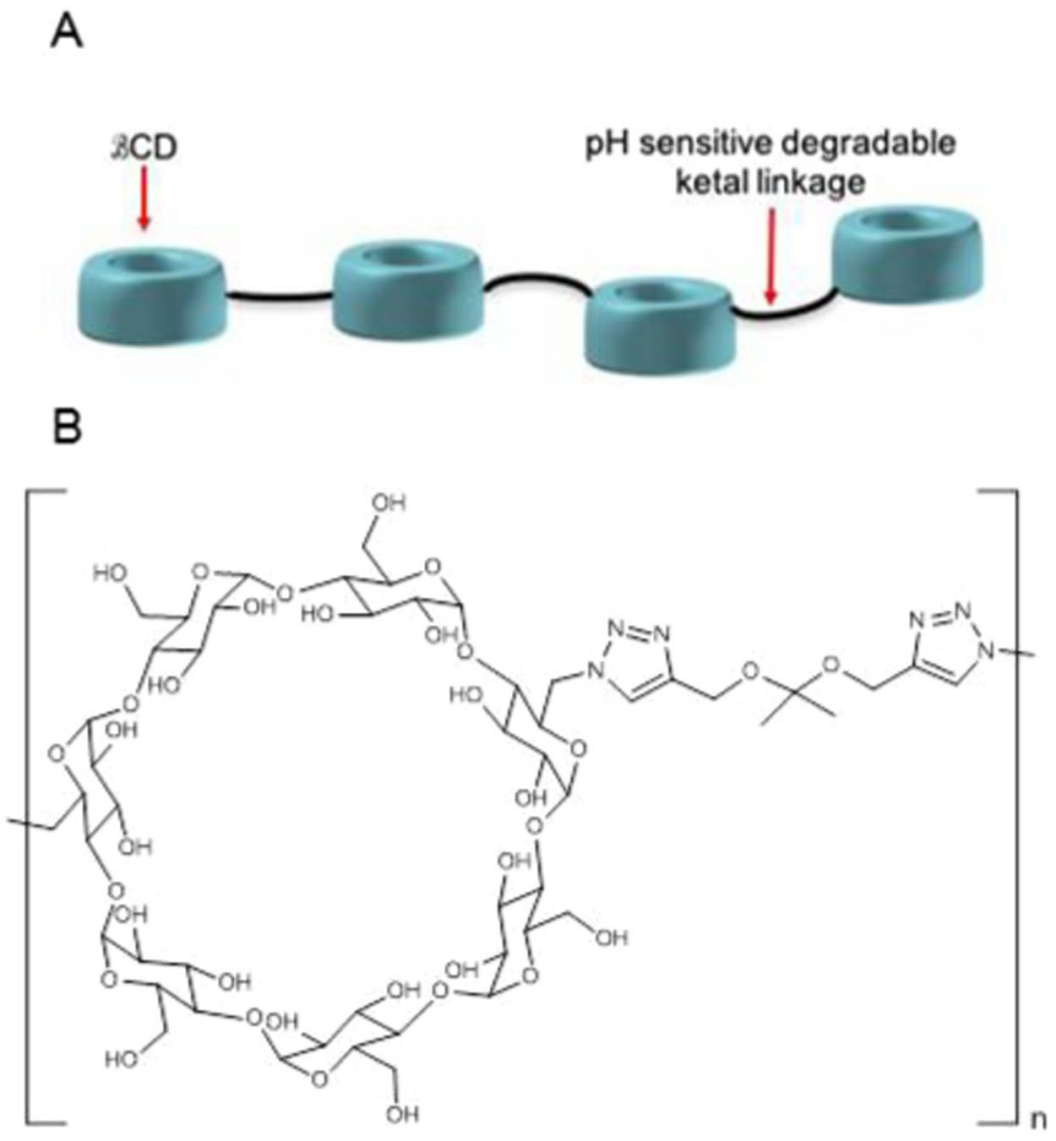
Schematic (**A**) and Chemical Structure (**B**) of ORX-301.

The strategy design and chemical structure of ORX-301 is displayed in Fig. 1, whereas a full description of its synthesis is provided in the Materials and Methods section of the Supplementary Information.

**Figure 2:**
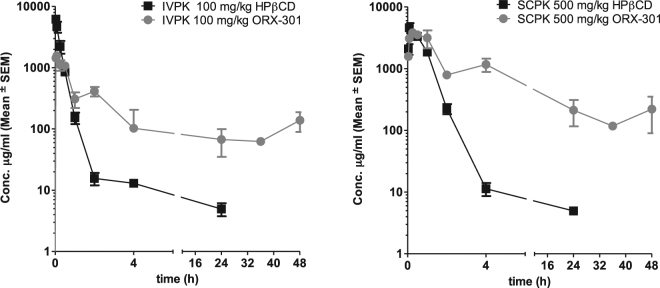
Comparative PK profiles of HPβCD v/s ORX-301 when administered intravenously at 100 mg/kg b.w. (left); and subcutaneously at 500 mg/kg b.w. (right) to BALB/c mice.

The pharmacokinetic profile of ORX-301 v/s HPβCD was evaluated in adult BALB/c mice after both intravenous (IV) - as well as subcutaneous (SC) -administrations (Fig. 2A,B). LC/MS/MS (HPβCD) and HPLC-UV (ORX-301) were used for analysis in the studies. The bioanalytical method for HPβCD was linear, accurate, and precise in the concentration range of 5.4 and 500 µg/ml, with an LLOQ = 5.4 µg/ml, and met all the acceptance criteria. The bioanalytical method for ORX-301 was linear, accurate, and precise in the concentration range of 13 and 500 µg/ml, with an LLOQ = 13 µg/ml, and met all the acceptance criteria.

Comparison of parameters such as t_max_, c_max_, AUC, and absolute bioavailability indicated that ORX-301 has a significantly improved profile over HPBCD with ORX-301 showing an absolute bioavailability of ~62% compared to 42% for HPβCD (Table 1).

**Table 1:** Comparison of PK parameters of HPβCD v/s ORX-301 when administered intravenous (100 mg/kg) or subcutaneously (500 mg/kg) to BALB/c mice.

Parameter	Intravenous		Subcutaneous	
	HPßCD	ORX-301	HPßCD	ORX-301
t_max_ (h)	0.083	0.25	0.083	0.25
c_max_ (µg/mL)	6198	1565	4613	3827
AUC_(0-t)_ [µg.h/mL]	1861	7151	4023	22179
AUC_(0-∞)_ [µg.h/mL]	1958	7274	4143	22777
Absolute Bioavailability (%)	42	62	42	62
t_1/2_ (h)	13.7	8.68	16.7	9.47
t_last_ (h)	24	48	24	48
c_last_ (µg/mL)	4.95	139	4.95	220.77

After a single intravenous (IV) dose of 100 mg/kg HPβCD showed a CL, V_ss_ and t_1/2_ of 0.051 l/h/kg, 0.18 l/kg and ~14 h, respectively and plasma levels were detectable only up to 24 h. After a single IV dose of 100 mg/kg ORX-301 showed an apparent CL and V_ss_ of 0.014 l/h/kg and 0.234 l/kg, respectively, and significant levels were detected up to 48 h at concentrations as high as 139 µg/mL. ORX-301 showed ~5-fold lower clearance when compared to HPβCD (Fig. 2A).

Following a single subcutaneous (SC) dose of 500 mg/kg, ORX-301 showed approximately 6-fold lower clearance and 5-fold higher exposure when compared to HPβCD. While most of HPβCD was cleared from plasma within the first few hours and was undetectable within 24 h, ORX-301 was detectable in the plasma in concentrations of 220 µg/mL up to 48 h (Fig. 2B). These studies further confirm that ORX-301 has an improved pharmacokinetic profile coupled with sustained elimination.

**Figure 3:**
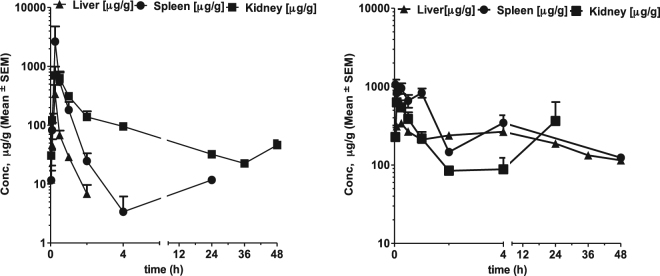
Distribution profiles of HPβCD (left) v/s ORX-301 (right) in the liver, spleen, and kidney when administered subcutaneously at 500 mg/kg b.w. to BALB/c mice.

The distribution profile of HPβCD and ORX-301 in the liver, kidney and spleen for a single SC administration of 500 mg/kg was also determined (Fig. 3, Table 2). High levels were observed in the kidney with an AUC_last_ = 2952 µg.h/ml for HPβCD and AUC_last_ = 5227 µg.h/ml. However, when compared with HPβCD [Liver AUC_last_ = 127 µg.h/ml; Spleen AUC_last_ = 828 µg.h/ml], the concentration of ORX-301 [Liver AUC_last_ = 8872 µg.h/ml; Spleen AUC_last_ = 11156 µg.h/ml] was found to be almost 70-fold greater in the liver, and 13-fold higher in the spleen.

**Table 2:** Comparison of distribution profile of HPβCD v/s ORX-301 in kidney, liver and spleen when administered subcutaneously (500 mg/kg) to BALB/c mice.

Organ	AUC_last_ [µg.h/mL]		Tissue/Plasma Ratio	
	HPßCD	ORX-301	HPßCD	ORX-301
Kidney	2952	5227	0.63	0.45
Liver	127	8872	0.03	0.76
Spleen	828	11156	0.18	0.95

**Figure 4:**
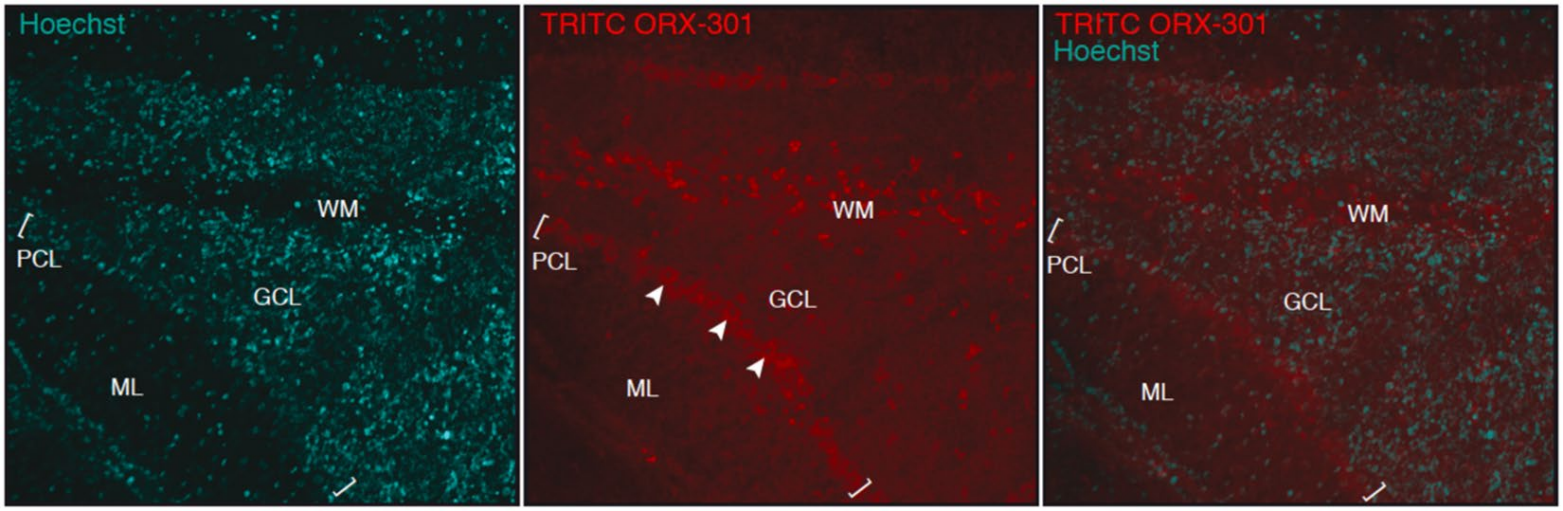
Visualization of TRITC-labeled ORX-301 in cerebellar sections of PN14 mice that had received a subcutaneous injection (1 mg/Kg in PBS, 50 µl total volume) 3 h before sacrifice. Arrowheads indicate TRITC-labeled ORX-301 positive Purkinje cells (PC). ML; molecular layer; GCL: granule cell layer; WM: white matter.

To ascertain whether ORX-301 was able to cross the BBB we administered TRITC-conjugated ORX-301 by a SC injection to 2-weeks-old BALB/C *wt* mice. After 3 h mice were sacrificed, brains were dissected and immediately frozen. Cryosections were then readily analyzed on a fluorescence microscopy, observing red-fluorescence staining of cerebellar cell bodies (Fig. 4). This confirmed that ORX-301 could penetrate the BBB when administered subcutaneously.

### Dose-range safety of ORX-301

Maximum Tolerated Dose (MTD) studies, performed on 6–8 weeks-old BALB/c *wt* mice, revealed that ORX-301 was safe up to a dose of 2000 mg/kg when administered subcutaneously. In fact, treated mice showed no clinical signs of toxicity and survived up to day 7, when they were sacrificed. Additionally, no adverse effects on body weight were observed (SI, Table 1A,B). Finally, gross necropsy did not reveal any pathological changes either (not shown). Instead, when administered with a higher dose (3000 mg/kg), the animals showed signs of dehydration (6/6 mice) and sluggish movement (3/3 male mice, 1/3 female mice); moreover, a hard mass was observed at the site of injection that persisted till the last day of study (day 7). Mortality of 1/3 male mice and 1/3 female mice was observed at this dose. The MTD was hence considered to be 2000 mg/kg.

### Administration of ORX-301 to *Npc1*^*nmf164/nmf164*^ mice improves their neurobehavioral phenotype and life span

Based on the improved biodistribution and pharmacokinetics of ORX-301 compared to HPβCD, we decided to investigate the efficacy of the ORX-301 prodrug at a dosage 5-fold lower than the routinely used HPβCD dosage (4000 mg/kg). Two studies were carried out to determine the efficacy of ORX-301 in Npc1- deficient mice. The first study was a late intervention model where ORX-301 was administered to *Npc1*^*nmf164/nmf164*^, hereafter referred to as *Npc1*^*nmf164*^, after the age of ~7 weeks, just before onset of symptoms. The second study was an early intervention model, where ORX-301 was administered to the *Npc1*^*nmf164*^ mice before weaning.

**Figure 5:**
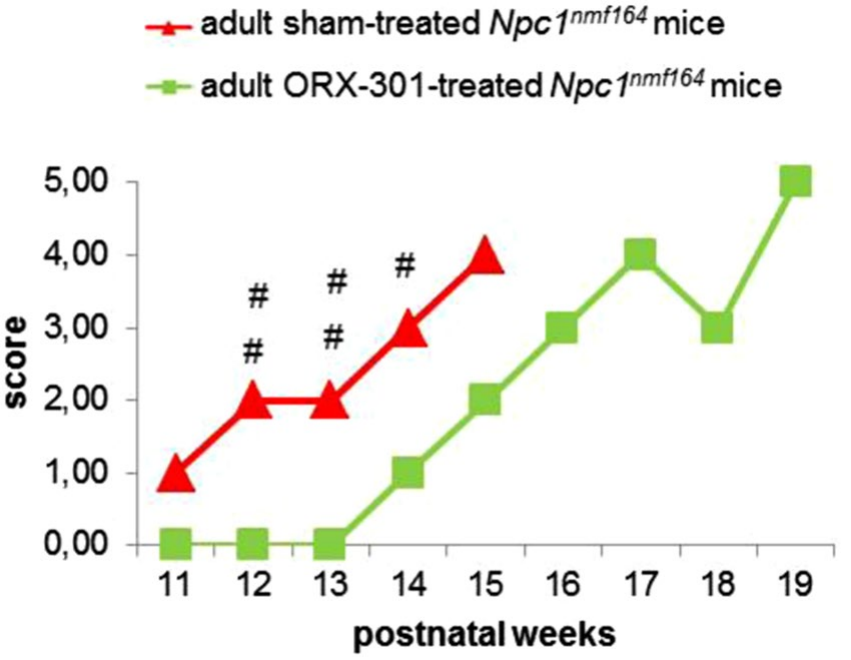
Administration of ORX-301 prodrug delays the body weight loss of adult pre-symptomatic *Npc1*^*nmf164*^ mice. Line graph indicates body weight loss scores of experimental group mice of increasing age. Data are expressed as median value. ORX-301- *vs*. sham-treated *Npc1*^*nmf164*^ mice: ^#^p < 0.05; ^##^p < 0.01.

In the late intervention model, we wanted to evaluate the ability of ORX-301 to counteract the progressive physical and neurological deterioration associated with Npc1 deficiency when administered to ~7 weeks old adult *Npc1*^*nmf164*^ mice. We first ruled out any dangerous effect of the treatment with 800 mg/kg ORX-301 by determining the body weight and neurobehavioral performance of ORX-301- and sham-treated *wt* adult mice, observing no significant difference (SI, Fig. 1 and Tables 2 and 3). Based on this finding, ORX-301- and sham-treated *wt* mice were pooled together and handled as a single group, hereafter named “*wt”* group. Thereafter, *Npc1*^*nmf164*^ mice were either sham- or ORX-301-treated at the aforementioned dose. To analyze the effect of ORX-301 treatment on body weight loss, we assigned to each mouse a score ranging from 0 to 5, with higher score corresponding to higher severity of weight loss (Table 3). We found that sham-treated *Npc1*^*nmf164*^ mice obtained higher scores at weeks 12–14 when compared to those obtained by ORX-301-treated mice, suggesting that ORX-301 treatment slowed the progression of body weight loss (Fig. 5; SI, Table 4).

**Table 3:** Scores used to assess body weight loss.

Score	Description
0	Weight loss up to 5%
1	Weight loss > 5 up to 10%
2	Weight loss > 10 up to 15%
3	Weight loss > 15 up to 20%
4	Weight loss > 20 up to 25%
5	Weight loss > 25 up to 30%

**Figure 6:**
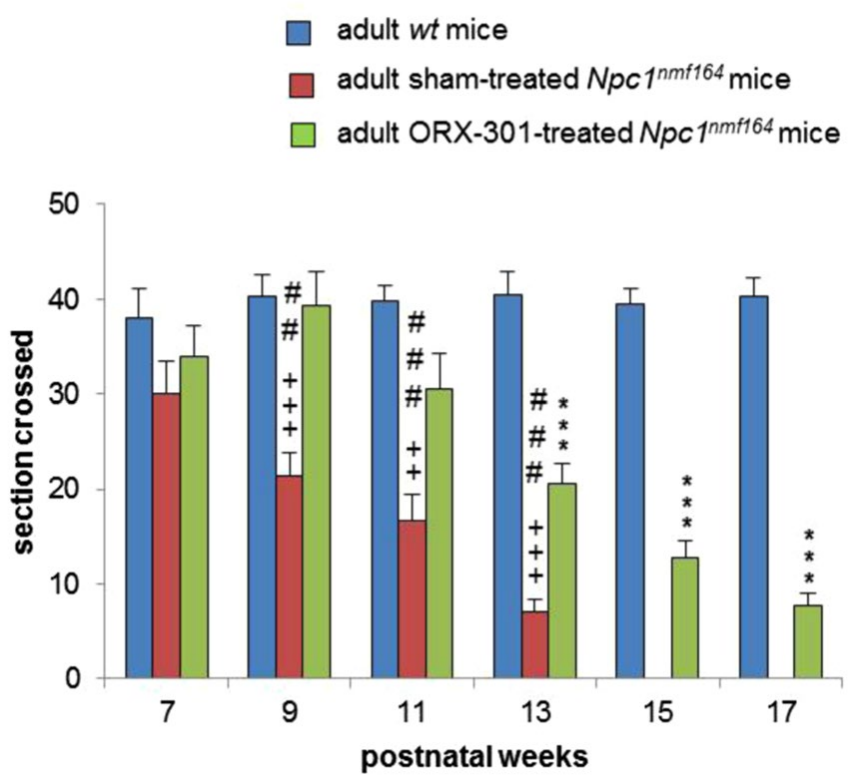
Administration of ORX-301 prodrug slightly improves neurobehavioral phenotype of adult pre-symptomatic *Npc1*^*nmf164*^ mice. Histogram indicates number of sections crossed by experimental group mice of increasing age, in the balance beam test. Data are expressed as mean ± SEM. *wt vs*. sham-treated *Npc1*^*nmf164*^ mice: ^+++^p < 0.001; *wt vs*. ORX-301-treated *Npc1*^*nmf164*^ mice: ***p < 0.001; ORX-301- *vs*. sham-treated *Npc1*^*nmf164*^ mice: ^##^p < 0.01; ^###^p < 0.001.

The treatment with ORX-301 also delayed the onset and slowed the progression of ataxic symptoms. In fact, ataxic symptoms were first detected in 9-weeks-old sham-treated *Npc1*^*nmf164*^ mice and in 13-weeks-old ORX-301-treated *Npc1*^*nmf164*^ mice, respectively. It was further encouraging that ORX-301-treated *Npc1*^*nmf164*^ mice did not differ from *wt* mice up to week 13 (Fig. 6; SI, Table 5).

**Figure 7:**
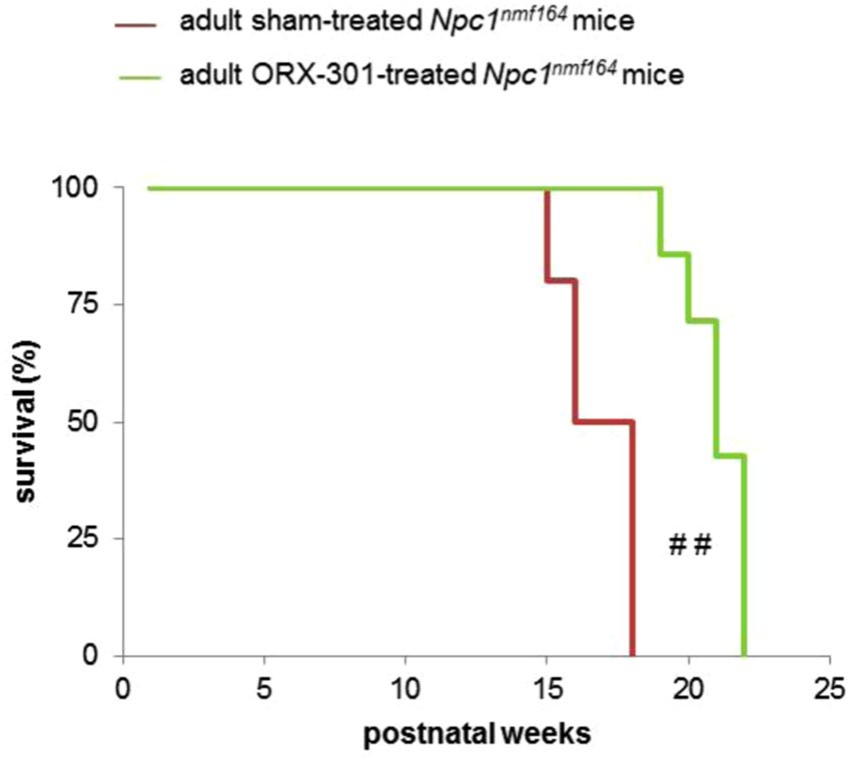
Administration of ORX-301 prodrug slightly improves life span of adult pre-symptomatic *Npc1*^*nmf164*^ mice. Kaplan-Meier plot of mouse survival. ORX-301- *vs*. sham-treated *Npc1*^*nmf164*^ mice: ^##^p < 0.01.

Finally, analysis of the survival curves revealed a significant difference between ORX-301- and sham-treated *Npc1*^*nmf164*^ mice (p = 0.002; Fig. 7). In fact, while all sham-treated *Npc1*^*nmf164*^ mice did not survive beyond the 17th week of age (mean lifespan: 114 ± 1.82 days), the treatment with ORX-301 significantly improved the lifespan, leading mice to survive up to the 21th week of age (mean lifespan: 140 ± 3.06 days), which corresponds to >20% improvement in survival.

In the second study, we wanted to evaluate the efficacy of ORX-301 when treatment was started in the mice of 2 weeks of age. To evaluate the safety and efficacy, both *wt* and *Npc1*^*nmf164*^ mice received weekly subcutaneous injections of 800 mg/kg ORX-301. The ORX-301 administration at this stage appeared well tolerated since it did not cause any detrimental effect on both physical growth and neurobehavioral performance of *wt* mice, which were perfectly comparable to those of sham-treated *wt* ones (SI, Fig. 2; Tables 6–7). Therefore, ORX-301- and sham-treated *wt* mice were pooled and handled as a single group, the “*wt*” group, similar to the late intervention study.

**Figure 8:**
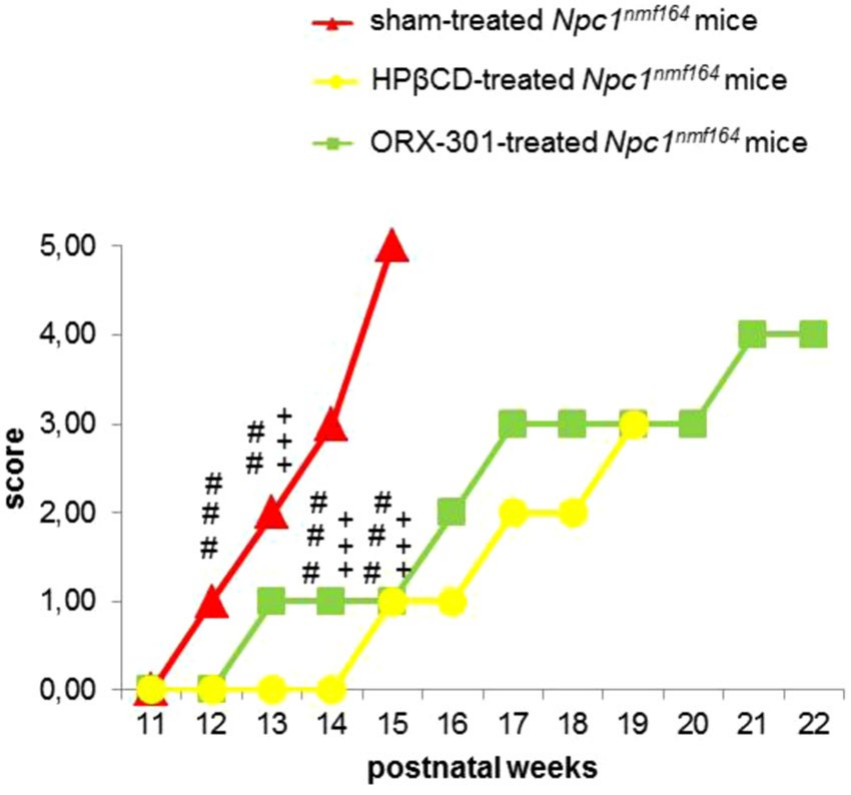
ORX-301 and HPβCD treatments delay progression of body weight loss in *Npc1*^*nmf164*^ mice at different rates when treated from PN14. Line graph indicates body weight loss scores of experimental group mice of increasing age. Data are expressed as median value. ORX-301- *vs*. sham-treated *Npc1*^*nmf164*^ mice: ^##^p < 0.01; ^###^p < 0.001; HPβCD vs. sham-treated *Npc1*^*nmf164*^ mice: ^+++^p < 0.001.

To demonstrate the improved efficacy of ORX-301 compared to HPβCD, an age-matched *Npc1*^*nmf164*^ group of mice that received 800 mg/kg HPβCD was included. Body weight measurements revealed that no significant treatment-dependent differences were observed in the various experimental groups (i.e. sham-, ORX-301-, HPβCD-treated groups) up to the age of 10 weeks (SI, Table 8). Thereafter, the body weight of sham-treated mice started to progressively decrease, as typically observed in naïve *Npc1*^*nmf164*^ mice^40^, whereas both ORX-301 and HPβCD-treated mice displayed a lower rate of weight loss progression. The severity of weight loss progression is indicated by scores (0–5) of the y-axis in Fig. 8. Weights of sham-treated *Npc1*^*nmf164*^ mice significantly differed from both ORX-301- and HPβCD-treated *Npc1*^*nmf164*^ mice starting from week 12 and 13, respectively (SI, Table 9). In fact, sham-treated *Npc1*^*nmf164*^ mice showed a noticeable and quite severe weight loss every week, HPβCD-treated *Npc1*^*nmf164*^ mice displayed a less severe and slower progressing weight loss, whereas ORX-301-treated *Npc1*^*nmf164*^ mice maintained a roughly constant body weight between days 91–112 and 126–140 (SI, Table 10).

**Figure 9:**
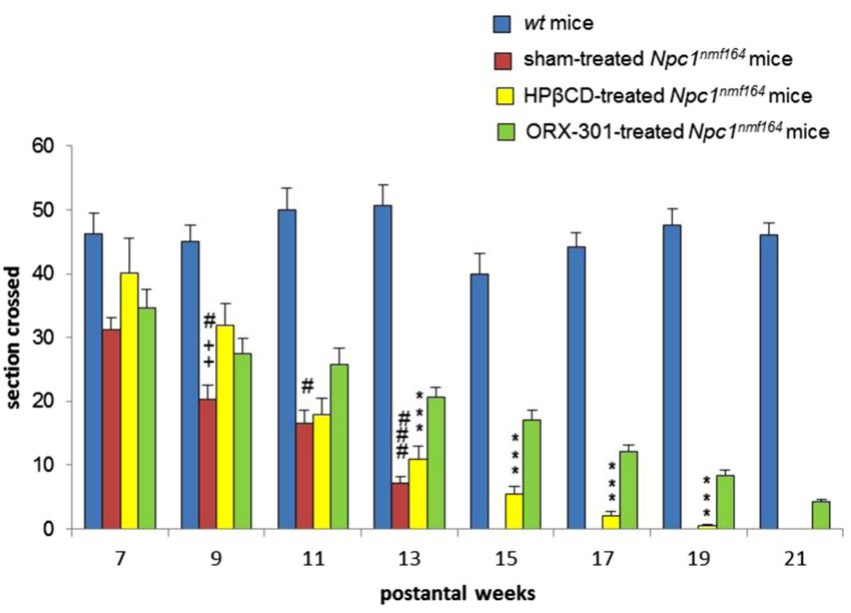
ORX-301 and HPβCD delay the onset of ataxic symptoms of *Npc1*^*nmf164*^ mice and ORX-301 significantly delays the progression of motor impairment. Histograms indicate the number of sections crossed by experimental group mice of increasing age in the balance beam test. Data are expressed as mean ± SEM. ORX-301 vs. sham-treated *Npc1*^*nmf164*^ mice: ^#^p 0.05, ^###^p < 0.001; HPβCD vs. sham-treated *Npc1*^*nmf164*^ mice: ^++^p < 0.01; ORX-301 vs. HPβCD-treated *Npc1*^*nmf164*^ mice: ***p < 0.001.

**Figure 10:**
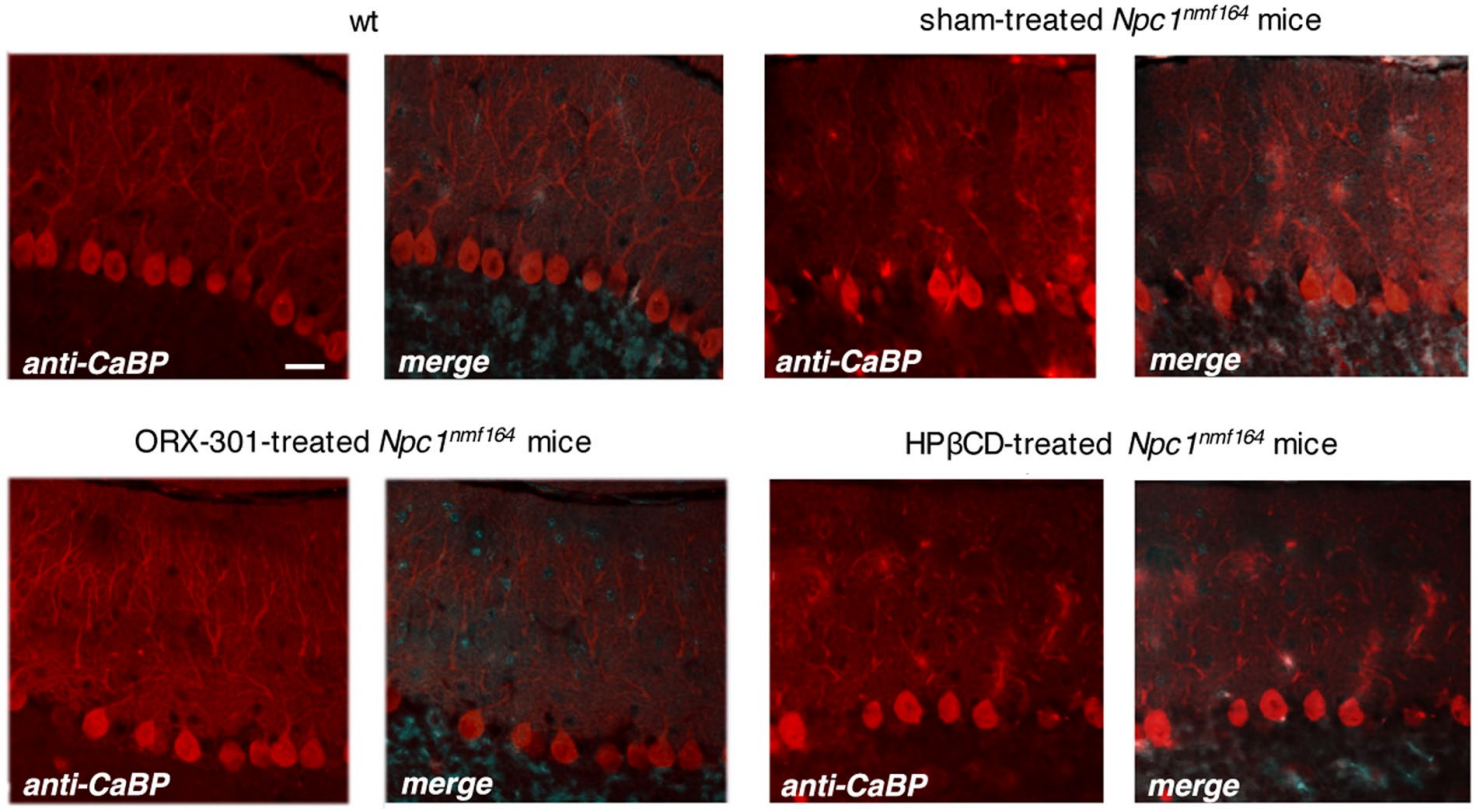
Immunostaining with antibodies directed to Calbindin (CaBP, red fluorescence) shows that *Npc1*^*nmf164*^ mice, either sham- or HPβCD-treated, display a reduced expression of Calbindin both in the soma and in dendritic tree compared to *wt* or ORX-301-treated ones. Nuclei were stained with Hoechst. Scale bar: 50 μm. Representative fields of parasagittal sections are shown.

Motor behavior studies demonstrated that both the ORX-301 and HPβCD treatments appeared to improve the performance of *Npc1*^*nmf164*^ mice in the balance beam test. This improvement in motor behavior is consistently observed in ORX-301-treated mice up to the end of the behavioral assessment, while the improvement associated with HPβCD-treatment is only temporarily observed up to 9-weeks of age (Fig. 9; SI Table 11). This observation is consistent with the ability of ORX-301 to counteract the impairment of Purkinje cell (PC) typical of *Npc1*-deficient mice. To verify this possibility we visualized the Ca^2+^-binding Calbindin protein on cerebellar sections observing that PCs of both sham- and HPβCD-treated 10 weeks-old *Npc1*^*nmf164*^ mice displayed a markedly reduced Calbindin staining compared to those of either *wt* or ORX-301-treated mice (Fig. 10). Noteworthy, Calbindin staining was barely detectable along the PC dendritic tree of sham- and HPβCD-treated *Npc1*^*nmf164*^ mice. Since Calbindin regulates the dynamics of post-synaptic Ca^2+^ gradients, thereby influencing motor coordination^41^, this observation is in agreement with the poor performance of these mice in the balance beam test (Fig. 9).

**Figure 11:**
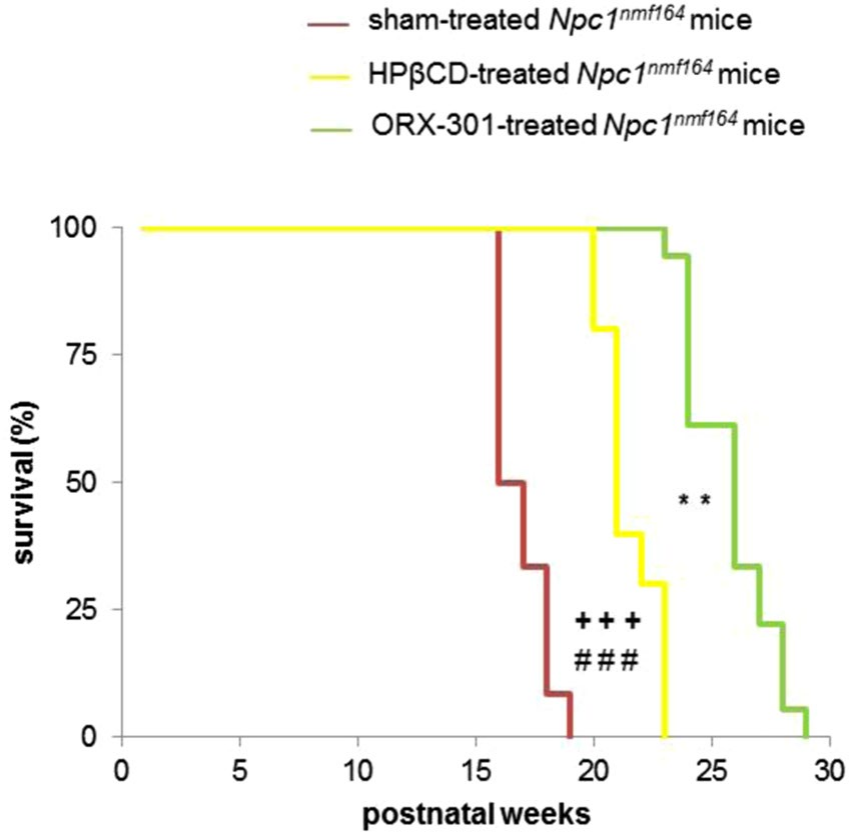
ORX-301 demonstrates better improvement in lifespan than HPβCD in *Npc1*^*nmf164*^ mice when administered subcutaneously at the same dose of 800 mg/kg. Kaplan-Meier plot of mouse survival. ORX-301 vs. sham-treated *Npc1*^*nmf164*^ mice: ^###^p < 0.001; HPβCD vs. sham-treated *Npc1*^*nmf164*^ mice: ^+++^p < 0.001; ORX-301 vs. HPβCD-treated *Npc1*^*nmf164*^ mice: **p < 0.01.

Finally, although both HPβCD and ORX-301 treatments improved the life span of *Npc1*^*nmf164*^ mice (p < 0.00001; Fig. 11), the ORX-301 prodrug displayed a superior efficacy. In detail, sham-treated *Npc1*^*nmf164*^ mice did not survive beyond day 120 (mean lifespan: 110 ± 2.19), HPβCD-treated *Npc1*^*nmf164*^ survived in average 150 ± 2.55 days, whereas ORX-301-treated Npc1nmf164 mice survived in average 170 ± 2.76 days.

## Discussion

The ORX-301 prodrug of this study represents a significant development in the context of available therapies for NPC disease, with particular reference to those based on cyclodextrins.

Cyclodextrin (CD) is a clinically approved excipient in the FDA’s list of Generally Regarded as Safe materials. CD and CD-based polymers are extensively used in the field of drug and gene delivery^42–45^. More recently, CD and its derivatives and especially HPβCD have shown efficacy in NPC as a potential therapeutic modality^46^. Preclinical studies in mouse and feline NPC models as well as the recent clinical trials have shown that HPβCD can reduce cholesterol accumulation in the affected organs, delay disease progression and extend survival^16–24,29^. However, HPβCD is faced with issues such as the need for high dose and intrathecal administration, inability to address visceral disease in current form of administration, and finally ototoxicity. Most of these issues are a result of the poor pharmacokinetic profile and bioavailability of HPβCD due to its propensity to undergo rapid renal elimination. In addition, due to the inability to penetrate the BBB, HPβCD is unable to address neurological symptoms unless administered intrathecally.

The findings of this study indicate that the degradable linear βCD-based polymer prodrug (ORX-301) overcomes these limitations, thereby validating the design strategy that its larger size would avoid the rapid renal filtration that is faced by and severely limits HPβCD efficacy. ORX-301 is a discrete linear polymer (low polydispersity index (PDI) of 1.021) of ~33,000 Da MW constructed from βCD monomer units linked together by a short degradable ketal linkage. We have demonstrated the synthesis of the ORX-301 polymer (MW ~ 33,000 Da, PDI 1.021) from βCD as a starting material. The polymer was synthesized in four steps based on previously reported procedures in high yields. The structure, size, and purity of ORX-301 were confirmed by 1 H NMR, GPC, and HPLC respectively.

Our hypothesis that the macromolecular design of ORX-301 would result in sustained elimination is fully confirmed by the findings of the present study. In fact, the pharmacokinetic studies we carried out in healthy BALB/c mice for both IV and SC administration showed that ORX-301 had a significantly improved bioavailability [62%] compared to HPβCD [42%], 5 to 6–fold lower clearance profile and 5–fold higher exposure. These studies further revealed that most of HPβCD is cleared within the first few hours and is almost undetectable at the end of 24 h which is in line with previous reports, whereas significant concentrations of ORX-301 [IV: 139 µg/mL; SC: 220 µg/mL] could be detected up to 48 h. Based on the high concentrations of both HPβCD and ORX-301 found in the kidney, it can be assumed that both compounds are largely eliminated through this organ, as indicated by previous reports for HPβCD^28^. The lower clearance and improved bioavailability are crucial aspects for lowering the dosage of the drug, still achieving the same therapeutic efficacy.

Secondly, tissue distribution determinations further showed that the accumulation of ORX-301 is almost 70-fold greater in the liver and 13-fold greater in the spleen than those observed with HPβCD. This data is extremely encouraging since HPβCD-based NPC treatment is unable to address the visceral disease in its current route of administration and ORX-301 can potentially address this issue.

Thirdly, a major issue faced by HPβCD is the need for intrathecal administration to address the neurological disease. This is because previous studies have shown that less than 0.2% of HPβCD reaches the brain upon systemic administration^25^. Our goal was to develop a cyclodextrin derivative that could potentially be administered subcutaneously or intravenously and still address the neurological disease. Upon subcutaneous administration of TRITC-labeled ORX-301 to 2-weeks-old mice, we observed the accumulation of the polymers within the cytoplasm of large neurons, such as Purkinje cells. We believe this can be partially attributed to the high cellular permeability of the polymers. This was also confirmed in a Caco-2 permeability assay carried out by us where it was seen that ORX-301 has a 4-fold higher permeability than HPβCD (data not shown). An additional contributing factor could be the improved pharmacokinetic and bioavailability profiles. While the rapid elimination of HPβCD may restrict the accumulation of the drug in the brain, in the case of ORX-301 the sustained elimination and higher bioavailability may allow for the higher systemic concentration of the drug to reach the brain. We hypothesize that a combination of these two factors accounts for the ORX-301 availability to the brain. Thereafter, membrane adsorption followed by endocytic engulfment likely allows the uptake of ORX-301 polymers by cells^47^.

Importantly, possible harmful effects of ORX-301 administration are ruled out by evidence provided by MTD studies performed in healthy BALB/c mice, which indicated that the drug was well tolerated at a dose up to 2000 mg/kg. In addition, the weekly chronic administration of ORX-301 (800 mg/kg) to either adult or pre-weaning *wt* mice had no detrimental effect on body weight and neurobehavioral performance, hence suggesting that subcutaneous weekly chronic administration of the drug at this dosage is safe.

Based on the combined pharmacokinetic and tissue distribution studies, BBB uptake evidence, and safety data, we decided to carry out efficacy studies in the *Npc1*^*nmf164*^ mouse model. These mice carry a single nucleotide change in the cysteine-rich luminal loop. This change confers a partial loss of activity to the NPC1 protein as observed in most common human mutations^40^. Compared to *Npc1*^*nih/nih*^ knockout mice, *Npc1*^*nmf164*^ mice display a milder phenotype and better mimic the large majority of human NPC cases, which include late-onset and slower progressing forms.

The first ORX-301 efficacy study we carried out was based on “a late intervention model” where adult pre-symptomatic mice (~7 weeks old) were treated with weekly doses of 800 mg/kg of ORX-301. Based on evidence previously provided by the phenotypic characterization of the *Npc1*^*nmf164*^ mouse model, this age is characterized by the appearance of typical signs of the disease, including the gradual body weight loss and motor performance decline^40^. The study revealed that ORX-301 could delay both body weight loss and motor decline by 4–5 weeks when compared with sham-treated mice. Additionally, the treatment also resulted in an extension in survival by almost 4 weeks amounting to an improvement in greater than 20%.

Although this improvement is quite small, it appears significant in light of contradicting evidence provided by studies addressing the efficacy of HPβCD treatment to counteract NPC disease progression in adult mice. Liu and coworkers reported that a single HPβCD injection at day 49 did not significantly improve the lifespan of *Npc1*^*nih/nih*^ mice, although it did reverse the cholesterol transport defect, indicating that the severe tissue damage of adult mice cannot be rescued by HPβCD administration^39^. On the other hand, Tanaka and co-workers demonstrated that weekly injections of HPβCD significantly enhanced the survival of *Npc1*^*nih/nih*^ mice, even when started at day 42^29^. The improvement appeared dose-dependent and required a dosage of at least 1000 mg/kg.

Previous studies showed that early postnatal HPβCD treatments, starting at day 7, resulted in a better efficacy on survival^39^ and also rescued developmental anomalies responsible for abnormal cerebellar morphogenesis^48,49^. Since HPβCD does not penetrate the BBB of the adult mouse brain, it is hypothesized that its beneficial effect, when administered to 7-days-old mouse pups, depends on a certain degree of leakiness of the BBB, which only acquires complete sealing properties later on^50^.

In our next study, we started the treatment with ORX-301 at day 14, when the formation of BBB is complete but cerebellar morphogenesis is still ongoing. This treatment schedule would allow ruling out a contribution of BBB leakiness to beneficial effects on neurological signs of the disease, while possibly rescuing neuronal and glial cell differentiation and synaptogenesis^50^. Hence, we hypothesized that the administration of ORX-301 to pre-weaning mice would result in much higher efficacy.

The inclusion of a cohort of sham-treated mice as a negative control and a cohort of mice treated with 800 mg/kg of HPβCD, among the experimental groups of this study, allowed us to gain a deeper insight on the therapeutic efficacy of ORX-301 polymers. Body weight measurements revealed that sham-treated mice had a rapid decline in their body weight, whereas both ORX-301 and HPβCD-treated mice displayed a lower rate of weight loss progression. However, although HPβCD-treated *Npc1*^*nmf164*^ mice displayed a less severe and slower progressing weight loss compared to sham-treated mice, the body weight of ORX-301-treated *Npc1*^*nmf164*^ mice did not significantly vary from wild-type during a quite large time interval. It is worth noting that during this time interval (days 91–140) sham-treated *Npc1*^*nmf164*^ undergo a significant worsening of their physical condition, including a rapid decrease of body weight. A similar beneficial effect was observed on motor performance, which was significantly and consistently improved by the treatment with ORX-301. By contrast, the treatment with HPβCD was associated with a temporary improvement of motor performance only at a significantly earlier mouse age. These findings are consistent with the beneficial effect on Purkinje cell function elicited by the treatment with ORX-301, as inferred by Calbindin immunostaining. Although still preliminary and deserving further investigation, this data strengthens the improved efficacy of ORX-301 over HPβCD.

Finally, with respect to the life span of *Npc1*^*nmf164*^ mice, the treatment with ORX-301 displayed a superior efficacy. Indeed, our findings demonstrated that at a dose of 800 mg/kg, ORX-301 could achieve almost 55% improvement in mean survival, whereas HPβCD at the same dose could achieve only 35% improvement. Previous studies reporting the efficacy of HPβCD at its full dose of 4000 mg/kg in *Npc1*^*nmf164*^ demonstrated an extension in mean lifespan up to 170–180 days^34^. This is comparable to the efficacy of ORX-301 at 1/5 the dose. Achieving a comparable efficacy at a lower dosage, which is likely not to affect the hearing threshold, represents a major advancement since a shortcoming of the efficacious HPβCD dosage is ototoxicity^27^.

Most importantly, since the synthesis of the polymer prodrugs is rapid, fairly straightforward and highly scalable, we believe that these molecules should not face any challenges with respect to their production while being translated into clinics.

## Conclusions

Taken together, results of this study indicate that our polymeric prodrug derivative of βCD, ORX-301, provides a promising therapeutic alternative for NPC1 disease. We have demonstrated that ORX-301 has comparable therapeutic efficacy to HPβCD despite being administered at 1/5 the dose. It also overcomes some of the major issues faced by HPβCD-based NPC treatment such has the high doses and a difficult route of administration. While, additional studies are required to better understand the effect of chronic administration on hearing along with dose escalation and dose frequency studies to identify the ideal dose regimen for optimal therapeutic efficacy, these studies provide strong proof-of-concept for our hypothesis thus warranting further evaluation.

## Methods

### Animals and treatments

*Npc1*^*nmf164*^ mice with BALB/cJ background obtained from heterozygous crosses were exposed to a 12 h light-dark cycle, receiving food and water ad libitum. Pup genotypes were identified by PCR analysis of tail DNA as previously described by Maue and coworkers^40^.

Pharmacokinetic analysis and MTD studies were carried out in BALB/c mice purchased from Taconic Biosciences. Protocols and procedures of these studies were approved by the Institutional Animal Ethics Committee based on CPCSEA (India) guidelines.

To determine the efficacy of ORX-301 prodrug *Npc1*^*nmf164*^ and age matched *wt* mice were subjected weekly to a subcutaneous injection of a 16% w/v ORX-301 solution in PBS (800 mg/kg body weight). Control group mice received plain PBS (sham, group). *Npc1*^*nmf164*^ and *wt* mice were randomly assigned to the various experimental groups, which had a comparable number of males and females, ruling out possible bias related to the sex.

In the late intervention study, *Npc1*^*nmf164*^ and *wt* mouse littermates were treated weekly with either ORX-301 or PBS starting from the 7th week of age. The body weight was recorded weekly and a score ranging from 0 to 5 was assigned to each mouse (Table 3) as previously performed^34^. The motor co-ordination phenotype was assessed by the balance beam test every two weeks as previously described^49^. Briefly, the mouse was placed perpendicularly at the center of a horizontal round beam (covered with paper tape, outer diameter 2 cm, length 1 m, divided into 10 sections and placed 50 cm above a padded surface) and the number of beam sections crossed in a 180 s time interval was recorded. Although the test relies on muscle strength and limb tone, it is well validated for the assessment of fine motor coordination and balance. In addition, as was previously demonstrated, the balance beam test is very sensitive to detect subtle motor impairment in *Npc1*^*nmf164*^ mice before the appearance of overt ataxic signs^49^. The end-point of data collection was established as mouse weight approaching 13 g^40^.

In the early intervention study, treatments were initiated in mice of 2 weeks of age and the efficacy of the ORX-301 prodrug was assessed along with that of a similar HPβCD concentration (16% w/v solution in PBS; 800 mg/kg body weight; Sigma Aldrich, Milan, Italy), by determining the life span, body weight loss and motor behavior.

Groups of *wt* and *Npc1*^*nmf164*^ mice were treated weekly with ORX-301 or HPβCD, as above (3 mice/genotype/treatment), from 2 weeks up to 10 weeks of age and then sacrificed to obtain brains for immunohistochemistry. Sham-treated control mice were injected with plain PBS.

Experimental protocols and related procedures of studies investigating the effect of ORX-301 and HPβCD on mouse survival and neurobehavioral phenotype were approved by the Italian Ministry of Public Health. All efforts were made to minimize animal suffering, according to European Directive 2010/63/EU.

### ORX-301 synthesis and characterization

Detailed procedures, associated analytical data and schemes of ORX-301 synthesis can be found in the Supplementary Information (SI, Materials and Methods, Scheme 1).

Briefly, the synthesis of the ORX-301 polymer was achieved by the polymerisation of βCD diazide monomer (3) and difunctionalized ketal linker (4) employing the 1,3 dipolar click reaction. The diazide monomer was synthesized according to reported procedure from βCD^51^. βCD was converted to the biphenyl-4,4′-disulfonate capped βCD (2) using biphenyl-4,4′-disulfonyl chloride. The intermediate (2), on treatment with dry potassium iodide in dry DMF under heating < gave diiodo βCD which further on treating with sodium azide in DMF under heating conditions afforded βCD diazide monomer (3) (SI, Scheme 1). The difunctionalized ketal linker was synthesized by treating (propargyloxy)trimethylsilane with acetone in the presence of a catalytic amount of trimethylsilyltrifluoromethanesulphonate in DCM at −78 °C resulting in the difunctionalized ketal linker (4) (SI, Scheme 1). Finally, ORX-301 was synthesized by the polymerization of the βCD diazide monomer (3) and difunctionalized ketal linker (4) (SI, Scheme 1). ORX-301 was characterized using NMR, GPC and HPLC. The molecular weight (MW) of the polymer determined by GPC (ELS detector) was found to be ~33 kDa with a PDI of 1.021. HPLC was carried out to determine if any of small molecule impurities were present in the polymer and it was found to be 100% pure.

A GPC based assay was carried out to determine the degradation profile of ORX-301 as a function of pH. The rate of ORX-301 degradation in DMF at 37 °C at pH 5.3 and 7.4 respectively was monitored by GPC/SEC (SI, Scheme 1). It was observed that ORX-301 degraded significantly faster at pH 5.3 compared to that at pH 7.4. This can be attributed to the ketal linkages that are pH labile. While the polymer degraded completely within 4 h at pH 5.3, only 70% was degraded at 24 h at pH 7.4, which is in line with our expectations.

### Pharmacokinetic and biodistribution analyses

The vehicle used for dissolution of test compounds was 0.9% Normal Saline. The required quantity of test compound (HPβCD and ORX-301) were weighed and transferred into vials to which appropriate volumes of 0.9% normal saline were added and vortexed to obtain uniform solutions of concentration of 100 mg/ml for the 500 mg/kg dose. The formulations were prepared on the day of the study. The test compounds were administered at 100 mg/kg for the IV study and 500 mg/kg for the SC study. For the tissue distribution study, again 500 mg/kg was administered by the SC route.

Post dose, blood, or the relevant tissue in the case of the distribution study was collected at time points of 2 min, 5 min, 15 min, 30 min, 1 h, 2 h, 4 h, 24 h, 36 h and 48 h. From each mouse terminal blood samples were collected by cardiac puncture under isoflurane anaesthesia. The blood was collected in 2 ml Eppendorf tubes containing 0.020 ml of 10% K2EDTA, mixed gently and placed in ice before centrifugation. Plasma was harvested and stored at −80 °C. HPβCD was quantified in plasma using a partially validated LCMS/MS methods while ORX-301 was quantified using a partially validated HPLC-UV method. The PK analysis was performed using Non-compartmental methods in Phoenix WinNonlin 6.4 [Pharsight, Certara]. The mean concentration-time data were used for computing the PK parameters. The AUC was computed using the linear up-log down method. The following NCA parameters were estimated for the IV PK: Systemic Clearance [CL], Vss [Volume of distribution at steady state], first order elimination rate constant [ke], terminal half-life [t1/2], AUC0-t, AUC0-∞. For the SC PK study: Cmax, tmax, ke, t1/2, AUC0-t, AUC0-∞ and the absolute SC bioavailability [F] was estimated using the equation:

$${\rm{F}}( \% )=(\mathrm{AUCsc}/\mathrm{AUCiv})\,\ast \,(\mathrm{Doseiv}/\mathrm{Dosesc})\,\ast \,{\rm{100}}$$

To determine the ability of the ORX-301 prodrug to cross the blood brain barrier (BBB), TRITC-conjugated ORX-301 (1 mg/kg body weight in PBS, 50 µl total volume) was subcutaneously administered to 2-weeks-old wt mice, which were then sacrificed after 3 h. Brains were readily dissected and sectioned (8 µm) to visualize the fluorescent staining.

### Maximum Tolerated Dose Studies

MTD studies were performed on adult BALB/c mice. Animals were acclimatized for a period of 7 days before experimentation. Healthy BALB/c mice (three animals/sex/dose) were administered the test item (ORX-301) at increasing doses (1000 mg/kg, 1500 mg/kg, 2000 mg/kg, 3000 mg/kg, and 4000 mg/kg body weight) by subcutaneous route prepared in 0.9% saline for injection at a dose volume of 10 mL/kg body weight. Animals of control group were administered the vehicle only. Once administered, animals were observed for 7 days and then sacrificed. Body weights were recorded on injection and sacrifice days. Additionally, animals were observed for mortality/morbidity and for any adverse clinical signs at 0.5 h, 1 h, 2 h and 4 h after the injection and then twice daily, up to 7 days.

### Calbindin detection by immunofluorescence

10-weeks-old *wt* and *Npc1*^*nmf164*^ mice either sham-, ORX-301-, or HPβCD-treated were anaesthetized by intraperitoneal injection of a mixture of xylazine/ketamine (20–34 mg/kg) and then transcardially perfused with PBS. Brains were quickly dissected, fixed overnight at 4 °C in 4% PFA and processed as previously described^52^. Briefly, fixed brains were dehydrated by incubation in 15–30% sucrose in PBS, embedded in FSC 22 Clear Frozen Section Compound (Leica Biosystems, Milano, Italy) and serially sectioned (slice thickness 10 µm) using a cryostat (Leica CM 1900). Sagittal sections were mounted on X-tra Adhesive glass slides (Leica biosystems). Sections were processed for immunofluorescence using a mouse monoclonal Calbindin antibody (Anti-Calbindin D28K, Sigma, Milan, Italy; 1:500 dilution in PBS) as routinely performed in our lab. A permeabilization step performed by incubating sections in 0.1% Triton X-100 in PBS for 10 min was followed by a 3 h incubation in a blocking solution containing 5% goat serum, 1% BSA, 0.2% Triton X-100 in PBS. Sections were then incubated over night with a 1:500 primary antibody dilution in PBS and, after several washes with PBS, with a TRITC-conjugated anti-mouse secondary antibody (Zymed-Thermo Fisher Scientific, Milan, Italy).

### Statistical Analyses

Statistical analyses were performed by using STATISTICA 8 (StatSoft). Data were first tested for normality (Wilk-Shapiro’s test) and homoscedasticity (Levene’s test), and then analyzed by unpaired two-tailed Student’s t test or one-way ANOVAs for independent (treatment or group) measures, followed by Bonferroni’s post-hoc test. When data did not fully meet parametric assumptions, or were ordinal (body weight loss), non-parametric analyses of variance (Kruskal-Wallis’s test followed by Mann-Whitney’s U test and Friedman’s test followed by Wilcoxon’s test) were used. Survival data were analyzed using the Kaplan-Meier method, and the log-rank test was used to compare statistical significances. Differences were considered significant at the p < 0.05 level.

## Electronic supplementary material

Supplementary Information
