# Supplementary material for: Linear Cyclodextrin Polymer Prodrugs as Novel Therapeutics for Niemann-Pick Type C1 Disorder
Source: Sci Rep. 2018 Jun 22;8:9547. doi: 10.1038/s41598-018-27926-9 (PMC6015065; doi:10.1038/s41598-018-27926-9)
Supplement: Supplementary file 1 — Supplementary Information [file 41598_2018_27926_MOESM1_ESM.docx]

Linear Cyclodextrin Polymer Prodrugs as Novel Therapeutics for Niemann-Pick Type C1 Disorder

Aditya Kulkarni,^1*^ Paola Caporali,^2^ Atul Dolas,^1^ Soniya Johny,^1^ Sandeep Goyal,^1^ Jessica Dragotto,^2^ Alberto Macone,^3^ Ramesh Jayaraman^4^ and Maria Teresa Fiorenza^2,5^

^1^Aten Porus Lifesciences, Bangalore 560068, India.

^2^Department of Psychology, Division of Neuroscience, Sapienza University, Rome, Italy.

^3^Department of Biochemical Sciences "A. Rossi Fanelli", Sapienza University of Rome Rome, Italy.

^4^TheraIndx Lifesciences Pvt Ltd, Bangalore 562123, India.

^5^IRCCS Fondazione Santa Lucia, Via del Fosso Fiorano 64, 00179 Rome, Italy.

*To whom correspondence should be addressed: aditya@atenporus.com

Supplementary Information

**Synthesis of ORX-301**

**Materials and Methods**

All the reagents were obtained from commercial sources and used as such without further purification if not specified. All solvents used for synthesis were of analytical grade and used without any further purification. IR spectra were measured with a Perkin Elmer Spectrum One Fourier transform infrared spectrometer as KBr pellets. ^1^H NMR spectra were recorded on a 400 MHz Bruker spectrometer using TMS as an internal standard. The chemical shifts (δ) for ^1^H are given in ppm relative to residual signals of the solvent. Coupling constants are given in Hz. The following abbreviations are used to indicate the multiplicity: s, singlet; d, doublet; t, triplet; q, quartet; m, multiplet; brs, broad singlet.

**Synthesis and Characterization**

*Synthesis of 6^A^, 6^D^-diazido 6^A^, 6^D^-dideoxy β-cyclodextrin (****3****)*

To a solution of 6^A^, 6^D^-diiodo 6^A^, 6^D^-dideoxy *β*-CD(8 g, 5.9 mmol, 1 equiv.) in anhydrous dimethylformamide (70.0 mL),NaN_3_ (2.3 g, 35.4 mmol, 6 equiv.) was added. The resulting solution was stirred at 80 °C for 14 h under nitrogen atmosphere. The solution was concentrated to remove DMF and the residue was dissolved in minimum amount of water and loaded on silica gel. The compound was then purified through column chromatography using ACN:water (95:5) as the eluent system. The appropriate fractions were combined together and evaporated to remove acetonitrile. The aqueous solution was then lyophilized to afford desired product as a white solid (Yield: 5.6 g, 80%). FT–IR (KBr, cm^-1^): 3366, 2037; ^1^HNMR (400 MHz, D_2_O) *δ =*5.04 (d, *J* = 3.6 Hz, 7H), 3.77-4.00 (m, 28H), 3.53-3.65 (m, 14H), 2.83 (s, 2H), 3.98 (s, 2H).

*Synthesis of difunctionalized ketal linker (****4****)*

To a solution of acetone (1.43 mL, 19.5 mmol, 1 equiv.) and (propargyloxy)trimethylsilane (5 gm, 38.9 mmol, 2.0 equiv.) in dry dichloromethane under inert atmosphere at -78 °C, trimethylsilyl trifluoromethanesulphonate (0.86 gm, 20 mol%) was added slowly. The reaction mixture was allowed to stir at same temperature for an additional 2.5 h. Progress of the reaction was monitored by TLC. Pyridine (0.6 mL) was added to the reaction mixture and allowed to stir for further 15 min. After completion of the reaction, the reaction mixture was poured in to the solution of saturated sodium bicarbonate and was extracted with diethyl ether. The collective organic layer was washed with brine and evaporated to dryness under reduced pressure. The crude residue was purified through column chromatography using hexane/EtOAc (95:05) as the eluent system to afford Compound **4** (2.2 g, 76%) as a colourless oil. ^1^HNMR (400 MHz, CDCl_3_):*δ* = 4.16 (d, *J* = 2.7 Hz, 4H), 2.41 (t, *J* = 2.4 Hz, 2H), 1.43 (s, 6H); ^13^C NMR (100 MHz, CDCl_3_):*δ =* 101.51, 80.46, 73.46, 49.36, 24.62.

*Synthesis of polymer (****ORX-301****)*

To a degassed solution of **3** (1 g, 0.84 mmol, 1 equiv.) in dimethylformamide (0.2 M), **4**(128 mg, 0.84 mmol, 1 equiv.) and Cu(PPh_3_)_3_Br (39 mg, 0.042 mmol, 0.05 equiv.) were added. The solution was stirred with heating at 60°C for 24 h. The viscous solution was poured into a large excess of ethyl acetate (10x of reaction volume) and washed with hexane. The resulting precipitate was filtered through Buchner funnel. The solid product obtained was dissolved in minimum amount of water and lyophilized to afford desired polymer (ORX-301).^1^H NMR (400 MHz, D_2_O) *δ* = 7.88 (s, 1H), 5.01 (d, *J* = 3.6 Hz, 7H), 3.53-3.93 (m, 47H), 2.96 (s, 3H), 2.80 (s, 3H). The molecular weight of the polymer is determined by gel permeation chromatography (GPC) in DMF. ORX-301 has a molecular weight peak (Mp) of ~ 33,000 Da and a polydispersity index (PDI) of 1.021. The purity of the ORX-301 was determined by HPLC.


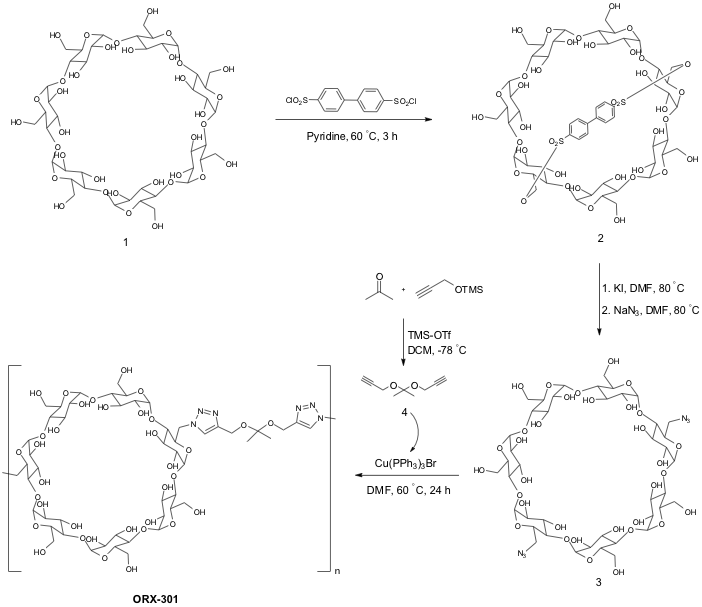


**Scheme 1**. Synthesis of *β*-cyclodextrin click polymer (ORX-301)

**Table 1A**: Individual Animal Body Weight- Males

| **Group No**  **Dose**  **(mg/kg b.w)** | **Animal**  **ID** | **Day 1** | **Day 7** |
| --- | --- | --- | --- |
| **G1**  **0** | **M001**  **M002**  **M003** | 18.60  20.51  19.92 | 19.88  20.38  20.34 |
| **G2**  **2000** | **M004**  **M005**  **M006** | 20.00  19.04  18.67 | 19.35  19.90  19.78 |
| **G3**  **3000** | **M007**  **M008**  **M009** | 20.59  19.69  19.87 | Dead  19.70  19.09 |

**Table 1B**: Individual Animal Body Weight- Females

| **Group No**  **Dose**  **(mg/kg b.w)** | **Animal**  **ID** | **Day 1** | **Day 7** |
| --- | --- | --- | --- |
| **G1**  **0** | **M013**  **M014**  **M015** | 17.96  18.65  18.13 | 17.99  18.78  18.90 |
| **G2**  **2000** | **M016**  **M017**  **M018** | 17.62  18.20  16.05 | 17.99  18.38  16.18 |
| **G3**  **3000** | **M019**  **M020**  **M021** | 18.13  17.84  19.37 | 18.10  17.75  Dead |

**Table 2:** Statistical analyses of the body weight of adult *wt* mice of increasing age

| **Post-natal**  **week** | **Mean bodyweight** | | **Mann-Whitney U tests**  **adult ORX-301- *vs.* sham-treated *wt* mice** |
| --- | --- | --- | --- |
|  | **adult sham-treated**  ***wt* mice** | **adult ORX-301-treated**  ***wt* mice** |  |
| 8 | 20.46 ± 0.83 g | 20.35 ± 0.65 g | U = 35.5, p = 0.70 |
| 9 | 20.83 ± 0.72 g | 20.91 ± 0.52 g | U = 33.5, p = 0.57 |
| 10 | 21.89 ± 0.82 g | 21.71 ± 0.68 g | U = 38, p = 0.90 |
| 11 | 21.98 ± 0.74 g | 22.07 ± 0.56 g | U = 33, p = 0.57 |
| 12 | 23.79 ± 0.84 g | 23.72 ± 0.61 g | U = 36, p = 0.76 |
| 13 | 24.34 ± 0.91 g | 24.58 ± 0.49 g | U = 37.5, p = 0.83 |
| 14 | 24.59 ± 0.85 g | 24.78 ± 0.47 g | U = 38, p = 0.90 |
| 15 | 25.23 ± 0.83 g | 25.18 ± 0.45 g | U = 34.5, p = 0.63 |
| 16 | 25.19 ± 0.83 g | 25.20 ± 0.41 g | U = 28, p = 0.31 |
| 17 | 25.40 ± 0.96 g | 25.65 ± 0.43 g | U = 33, p = 0.57 |
| 18 | 25.29 ± 0.89 g | 25.14 ± 0.53 g | U = 34.5, p = 0.63 |
| 19 | 25.92 ± 0.93 g | 26.21 ± 0.36 g | U = 37, p = 0.83 |
| 20 | 26.19 ± 0.85 g | 25.82 ± 0.55 g | U = 34, p = 0.63 |
| 21 | 25.87 ± 1.09 g | 26.33 ± 0.50 g | U = 38, p = 0.90 |


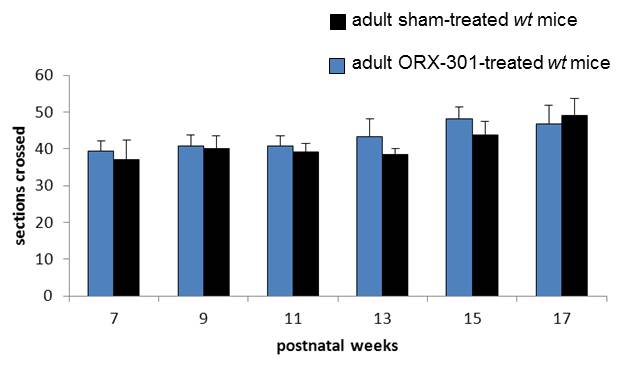


**Figure 1.** The administration of the ORX-301 prodrug has no effect on the neurobehavioral phenotype of adult *wt* mice. Histograms indicate the number of sections crossed by experimental group mice of increasing age in the balance beam test. Data are expressed as mean ± SEM.

**Table 3:** Statistical analyses of the neurobehavioral performance of adult *wt* mice of increasing age

| **Post-natal**  **week** | **Student’s t test**  **adult ORX-301- *vs.* sham-treated *wt* mice** |
| --- | --- |
| 7 | t_16_ = - 0.36, p = 0.73 |
| 9 | t_16_ = - 0.18, p = 0.86 |
| 11 | t_16_ = - 0.45, p = 0.61 |
| 13 | t_16_ = - 1.04, p = 0.31 |
| 15 | t_16_ = - 0.86, p = 0.40 |
| 17 | t_16_ = 0.34, p = 0.74 |

**Table 4:** Statistical analyses of the body weight loss variation of adult *Npc1^nmf164^* mice of increasing age.

| **Post-natal**  **week** | **Mann-Whitney U tests**  **adult ORX-301- *vs.* sham-treated *Npc1^nmf164^* mice** |
| --- | --- |
| 11 | U = 27.5, p = 0.47 |
| 12 | U = 8, p = 0.007 |
| 13 | U = 8, p = 0.007 |
| 14 | U = 13, p = 0.03 |
| 15 | U = 15.5, p = 0.06 |

**Table 5:** Statistical analyses of the neurobehavioral performance of adult mice of increasing age

| **Post-natal**  **week** | **Statistical analyses** | | | |
| --- | --- | --- | --- | --- |
| 7 | **one-way ANOVAs** | F_(2,32)_ = 1.48, p = 0.24 | *post hoc* | ------ |
| 9 |  | F_(2,32)_ = 14.60, p = 0.00003 | *post hoc* | adult *wt* *vs.* sham-treated *Npc1^nmf164^* mice: p = 0.00003 |
|  |  |  |  | adult *wt* *vs.* ORX-301-treated *Npc1^nmf164^* mice: p = 1 |
|  |  |  |  | adult ORX-301- *vs.* sham-treated *Npc1^nmf164^* mice: p = 0.001 |
| 11 |  | F_(2,32)_ = 25.12, p < 0.00001 | *post hoc* | adult *wt* *vs.* sham-treated *Npc1^nmf164^* mice: p < 0.00001 |
|  |  |  |  | adult *wt* *vs.* ORX-301-treated *Npc1^nmf164^* mice: p = 0.06 |
|  |  |  |  | adult ORX-301- *vs.* sham-treated *Npc1^nmf164^* mice: p = 0.005 |
| 13 |  | F_(2,32)_ = 58.17, p < 0.00001 | *post hoc* | adult *wt* *vs.* sham-treated *Npc1^nmf164^* mice: p < 0.00001 |
|  |  |  |  | adult *wt* *vs.* ORX-301-treated *Npc1^nmf164^* mice: p = 0.00001 |
|  |  |  |  | adult ORX-301- *vs.* sham-treated *Npc1^nmf164^* mice: p = 0.005 |
| 15 | **Mann-Whitney U** | adult *wt* *vs.* ORX-301-treated *Npc1^nmf164^* mice: U = 0, p < 0.00001 | | |
| 17 |  | adult *wt* *vs.* ORX-301-treated *Npc1^nmf164^* mice: U = 0, p < 0.00001 | | |

**Table 6:** Statistical analyses of the body weight of *wt* mice of increasing age

| **Post-natal**  **week** | **Mean bodyweight** | | **Mann-Whitney U test**  **ORX-301- *vs.* sham-treated *wt* mice** |
| --- | --- | --- | --- |
|  | **sham-treated**  ***wt* mice** | **ORX-301-treated**  ***wt* mice** |  |
| 2 | 7.89 ± 0.27 g | 7.35 ± 0.16 g | U = 30, p = 0.18 |
| 3 | 10.94 ± 0.24 g | 10.57 ± 0.26 g | U = 32, p = 0.24 |
| 4 | 13.99 ± 0.27 g | 13.78 ± 0.49 g | U = 43.5, p = 0.73 |
| 5 | 17.05 ± 0.35 g | 17.00 ± 0.74 g | U = 46.5, p = 0.91 |
| 6 | 19.15 ± 0.59 g | 18.60 ± 0.68 g | U = 37, p = 0.43 |
| 7 | 20.32 ± 0.67 g | 19.41 ± 0.86 g | U = 35, p = 0.34 |
| 8 | 21.36 ± 0.77 g | 20.23 ± 0.88 g | U = 32, p = 0.24 |
| 9 | 21.85 ± 0.76 g | 21.14 ± 0.95 g | U = 39, p = 0.52 |
| 10 | 22.78 ± 0.78 g | 21.46 ± 0.74 g | U = 33.5, p = 0.27 |
| 11 | 22.94 ± 0.84 g | 22.58 ± 0.85 g | U = 42, p = 0.68 |
| 12 | 23.34 ± 0.80 g | 22.89 ± 1.05 g | U = 37, p = 0.43 |
| 13 | 24.04 ± 0.79 g | 22.65 ± 1.12 g | U = 28, p = 0.14 |
| 14 | 24.75 ± 0.78 g | 23.02 ± 1.07 g | U = 27, p = 0.11 |
| 15 | 24.80 ± 0.56 g | 23.81 ± 0.62 g | U = 35, p = 0.34 |
| 16 | 24.95 ± 0.75 g | 24.73 ± 0.93 g | U = 44, p = 0.79 |
| 17 | 24.99 ± 0.89 g | 23.92 ± 0.95 g | U = 32, p = 0.24 |
| 18 | 25.10 ± 0.68 g | 24.61 ± 0.32 g | U = 41, p = 0.62 |
| 19 | 25.56 ± 0.74 g | 24.35 ± 0.98 g | U = 39, p = 0.52 |
| 20 | 25.16 ± 0.98 g | 25.43 ±1.10 g | U = 44, p = 0.79 |
| 21 | 25.31 ± 0.78 g | 25.83 ± 0.81 g | U = 40, p = 0.57 |
| 22 | 25.56 ± 0.61 g | 26.01 ± 0.78 g | U = 41, p = 0.62 |
| 23 | 25.78 ± 0.59 g | 26.35 ± 0.28 g | U = 32, p = 0.24 |
| 24 | 25.89 ± 1.01 g | 26.50 ± 0.55 g | U = 30, p = 0.18 |
| 25 | 25.91 ± 0.59 g | 26.58 ± 0.80 g | U = 30, p = 0.18 |
| 26 | 25.58 ± 0.63 g | 26.45 ±1.15 g | U = 30, p = 0.18 |
| 27 | 25.99 ± 0.77 g | 26.51 ± 0.95 g | U = 30, p = 0.18 |
| 28 | 26.16 ± 0.66 g | 26.60 ± 1.02 g | U = 30, p = 0.18 |

**
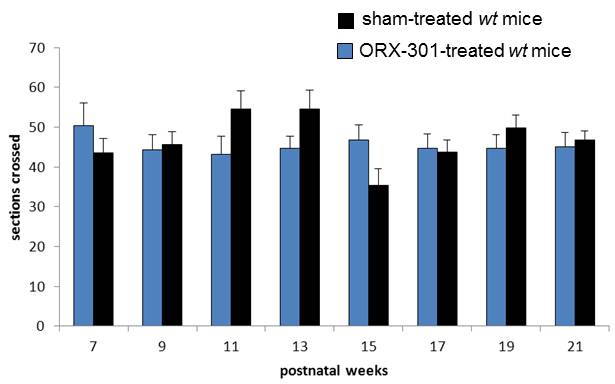
**

**Figure 2:** The administration of the ORX-301 prodrug has no effect on the neurobehavioral phenotype of *wt* mice. Histograms indicate the number of sections crossed by experimental group mice of increasing age in the balance beam test. Data are expressed as mean ± SEM.

**Table 7:** Statistical analyses of the neurobehavioral performance of *wt* mice of increasing age

| **Post-natal**  **week** | **Student’s t test**  **ORX-301- *vs.* sham-treated *wt* mice** |
| --- | --- |
| 7 | t_18_ = 1.06, p = 0.31 |
| 9 | t_18_ = - 0.24, p = 0.82 |
| 11 | t_18_ = - 1.70, p = 0.11 |
| 13 | t_18_ = - 1.56, p = 0.17 |
| 15 | t_18_ = 1.93, p = 0.07 |
| 17 | t_18_ = 0.17, p = 0.88 |
| 19 | t_18_ = - 1.05, p = 0.31 |
| 21 | t_18_ = - 0.43, p = 0.67 |

**Table 8:** Statistical analyses of the body weight of *Npc1^nmf164^* mice of increasing age

| **Post-natal**  **week** | **Mean bodyweight** | | | **Kruskal-Wallis’s**  **test** |
| --- | --- | --- | --- | --- |
|  | **sham-treated**  ***Npc1^nmf164^* mice** | **HPβCD-treated**  ***Npc1^nmf164^* mice** | **ORX-301-treated**  ***Npc1^nmf164^* mice** |  |
| 2 | 7.31 ± 0.20 g | 7.43 ± 0.30 g | 7.53 ± 0.21 g | H = 0.13, p = 0.94 |
| 3 | 10.44 ± 0.17 g | 10.37 ± 0.33 g | 10.02 ± 0.44 g | H = 0.98, p = 0.60 |
| 4 | 13.57 ± 0.33 g | 13.31 ± 0.53 g | 12.52 ± 0.34 g | H = 4.26, p = 0.12 |
| 5 | 16.48 ± 0.32 g | 17.24 ± 0.65 g | 15.76 ± 0.66 g | H = 1.40, p = 0.50 |
| 6 | 17.32 ± 0.37 g | 18.58 ± 0.75 g | 18.49 ± 0.64 g | H = 3.08, p = 0.21 |
| 7 | 18.09 ± 0.48 g | 20.25 ± 0.84 g | 19.97 ± 0.58 g | H = 0.93, p = 0.23 |
| 8 | 18.93 ± 0.59 g | 20.85 ± 0.94 g | 20.73 ± 0.37 g | H = 0.95, p = 0.48 |
| 9 | 19.94 ± 0.55 g | 20.55 ± 1.03 g | 19.96 ± 0.61 g | H = 0.21, p = 0.90 |
| 10 | 19.63 ± 0.67 g | 19.92 ± 0.81 g | 19.38 ± 0.55 g | H = 0.17, p = 0.92 |

**Table 9:** Statistical analyses of the body weight loss variation of *Npc1^nmf164^* mice of increasing age

| **Post-natal**  **week** | **Kruskal-Wallis’s**  **test** | **Mann-Whitney U test** | |
| --- | --- | --- | --- |
| 11 | H = 2.28, p = 0.32 | -------------------- | |
| 12 | H = 17.93, p = 0.0001 | ORX-301- *vs.* sham-treated *Npc1^nmf164^* mice | U = 22, p = 0.0001 |
|  |  | HPβCD- *vs.* sham-treated *Npc1^nmf164^* mice | U = 34, p = 0.09 |
|  |  | ORX-301- *vs.* HPβCD-treated *Npc1^nmf164^* mice | U = 58, p = 0.13 |
| 13 | H = 15.02, p = 0.0005 | ORX-301- *vs.* sham-treated *Npc1^nmf164^* mice | U = 42.5, p = 0.004 |
|  |  | HPβCD- *vs.* sham-treated *Npc1^nmf164^* mice | U = 7.5, p = 0.0001 |
|  |  | ORX-301- *vs.* HPβCD-treated *Npc1^nmf164^* mice | U = 66, p = 0.26 |
| 14 | H = 20.61, p < 0.00001 | ORX-301- *vs.* sham-treated *Npc1^nmf164^* mice | U = 16, p = 0.0002 |
|  |  | HPβCD- *vs.* sham-treated *Npc1^nmf164^* mice | U = 5.5, p = 0.00006 |
|  |  | ORX-301- *vs.* HPβCD-treated *Npc1^nmf164^* mice | U = 70.5, p = 0.36 |
| 15 | H = 25.70, p < 0.00001 | ORX-301- *vs.* sham-treated *Npc1^nmf164^* mice | U = 0, p < 0.00001 |
|  |  | HPβCD- *vs.* sham-treated *Npc1^nmf164^* mice | U = 0, p < 0.00001 |
|  |  | ORX-301- *vs.* HPβCD-treated *Npc1^nmf164^* mice | U = 78, p = 0.59 |
| 16 | ----- | ORX-301- *vs.* HPβCD-treated *Npc1^nmf164^* mice | U = 84.5, p = 0.80 |
| 17 | ----- | ORX-301- *vs.* HPβCD-treated *Npc1^nmf164^* mice | U = 68.5, p = 0.31 |
| 18 | ----- | ORX-301- *vs.* HPβCD-treated *Npc1^nmf164^* mice | U = 53.5, p = 0.08 |
| 19 | ----- | ORX-301- *vs.* HPβCD-treated *Npc1^nmf164^* mice | U = 89.5, p = 0.98 |

**Table 10:** Statistical analyses of the body weight loss variation of *Npc1^nmf164^* mice of increasing age

|  | | ***Npc1^nmf164^* mice** | | |
| --- | --- | --- | --- | --- |
|  |  | **sham-treated** | **HPβCD-treated** | **ORX-301-treated** |
| **Friedman’s Test** | | χ^2^ = 44.28, p < 0.00001 | χ^2^ = 38.14, p = 0.00001 | χ^2^ = 161.51, p < 0.00001 |
| **Wilcoxon’s test** | post-natal weeks 11 *vs.* 12 | Z = 2.80, p = 0.005 | Z = 0.67, p = 0.50 | Z = 0.91, p = 0.36 |
|  | post-natal weeks 12 *vs.* 13 | Z = 2.40, p = 0.02 | Z = 1.09, p = 0.27 | Z = 2.67, p = 0.008 |
|  | post-natal weeks 13 *vs.* 14 | Z = 2.52, p = 0.01 | Z = 1.28, p = 0.20 | Z = 1.78, p = 0.07 |
|  | post-natal weeks 14 *vs.* 15 | Z = 2.67, p = 0.008 | Z = 0.67, p = 0.50 | Z = 0.05, p = 0.96 |
|  | post-natal weeks 15 *vs.* 16 | ------ | Z = 0.94, p = 0.34 | Z = 0.05, p = 0.96 |
|  | post-natal weeks 16 *vs.* 17 | ------ | Z = 1.18, p = 0.24 | Z = 2.86, p = 0.004 |
|  | post-natal weeks 17 *vs.* 18 | ------ | Z = 0.52, p = 0.60 | Z = 2.45, p = 0.01 |
|  | post-natal weeks 18 *vs.* 19 | ------ | Z = 2.52, p = 0.01 | Z = 0, p = 1 |
|  | post-natal weeks 19 *vs.* 20 | ------ | ------ | Z = 1.83, p = 0.07 |
|  | post-natal weeks 20 *vs.* 21 | ------ | ------ | Z = 2.03, p = 0.04 |
|  | post-natal weeks 21 *vs.* 22 | ------ | ------ | Z = 0.36, p = 0.72 |

**Table 11:** Statistical analyses of the neurobehavioral performance of mice of increasing age

| **Postnatal**  **week** | **Kruskal-Wallis’s**  **test** | **Mann-Whitney U test** | |
| --- | --- | --- | --- |
| 7 | H = 13.35,  p = 0.004 | *wt* *vs.* sham-treated *Npc1^nmf164^* mice | U = 33, p = 0.0004 |
|  |  | *wt* *vs.* ORX-301-treated *Npc1^nmf164^* mice | U = 83, p = 0.004 |
|  |  | *wt* *vs.* HPβCD-treated *Npc1^nmf164^* mice | U = 85, p = 0.53 |
|  |  | ORX-301- *vs.* sham-treated *Npc1^nmf164^* mice | U = 94.5, p = 0.57 |
|  |  | HPβCD- *vs.* sham-treated *Npc1^nmf164^* mice | U =34, p = 0.09 |
|  |  | ORX-301- *vs.* HPβCD-treated *Npc1^nmf164^* mice | U = 68, p = 0.31 |
| 9 | H = 28.83,  p < 0.00001 | *wt* *vs.* sham-treated *Npc1^nmf164^* mice | U = 9.5, p < 0.00001 |
|  |  | *wt* *vs.* ORX-301-treated *Npc1^nmf164^* mice | U = 47.5, p = 0.00004 |
|  |  | *wt* *vs.* HPβCD-treated *Npc1^nmf164^* mice | U = 35, p = 0.004 |
|  |  | ORX-301- *vs.* sham-treated *Npc1^nmf164^* mice | U = 61, p = 0.04 |
|  |  | HPβCD- *vs.* sham-treated *Npc1^nmf164^* mice | U = 21.5, p = 0.009 |
|  |  | ORX-301- *vs.* HPβCD-treated *Npc1^nmf164^* mice | U = 59.5, p = 0.15 |
| 11 | H = 35.06,  p < 0.00001 | *wt* *vs.* sham-treated *Npc1^nmf164^* mice | U = 4.5, p < 0.00001 |
|  |  | *wt* *vs.* ORX-301-treated *Npc1^nmf164^* mice | U = 35.5, p < 0.00001 |
|  |  | *wt* *vs.* HPβCD-treated *Npc1^nmf164^* mice | U = 4, p < 0.00001 |
|  |  | ORX-301- *vs.* sham-treated *Npc1^nmf164^* mice | U = 52.5, p = 0.02 |
|  |  | HPβCD- *vs.* sham-treated *Npc1^nmf164^* mice | U =49, p = 0.50 |
|  |  | ORX-301- *vs.* HPβCD-treated *Npc1^nmf164^* mice | U = 57.5, p = 0.12 |
| 13 | H = 48.28,  p = 0.004 | *wt* *vs.* sham-treated *Npc1^nmf164^* mice | U = 0, p < 0.00001 |
|  |  | *wt* *vs.* ORX-301-treated *Npc1^nmf164^* mice | U = 10, p < 0.00001 |
|  |  | *wt* *vs.* HPβCD-treated *Npc1^nmf164^* mice | U = 0, p < 0.00001 |
|  |  | ORX-301- *vs.* sham-treated *Npc1^nmf164^* mice | U = 5.5, p < 0.00001 |
|  |  | HPβCD- *vs.* sham-treated *Npc1^nmf164^* mice | U =31.5, p = 0.06 |
|  |  | ORX-301- *vs.* HPβCD-treated *Npc1^nmf164^* mice | U = 26, p = 0.001 |
| 15 | H = 34.01,  p < 0.00001 | *wt* *vs.* ORX-301-treated *Npc1^nmf164^* mice | U = 26.5, p < 0.00001 |
|  |  | *wt* *vs.* HPβCD-treated *Npc1^nmf164^* mice | U = 1, p < 0.00001 |
|  |  | ORX-301- *vs.* HPβCD-treated *Npc1^nmf164^* mice | U = 12, p = 0.00004 |
| 17 | H = 40.49,  p < 0.00001 | *wt* *vs.* ORX-301-treated *Npc1^nmf164^* mice | U = 0, p < 0.00001 |
|  |  | *wt* *vs.* HPβCD-treated *Npc1^nmf164^* mice | U = 0, p < 0.00001 |
|  |  | ORX-301- *vs.* HPβCD-treated *Npc1^nmf164^* mice | U = 2, p < 0.00001 |
| 19 | H = 40.87,  p < 0.00001 | *wt* *vs.* ORX-301-treated *Npc1^nmf164^* mice | U = 0, p < 0.00001 |
|  |  | *wt* *vs.* HPβCD-treated *Npc1^nmf164^* mice | U = 0, p < 0.00001 |
|  |  | ORX-301- *vs.* HPβCD-treated *Npc1^nmf164^* mice | U = 0, p < 0.00001 |
| 21 | ---- | *wt* *vs.* ORX-301-treated *Npc1^nmf164^* mice | U = 0, p < 0.00001 |


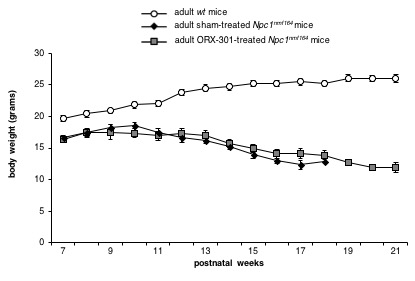


**Figure 3:** Administration of ORX-301 prodrug delays body weight loss of adult pre-symptomatic (late-intervention study) *Npc1^nmf164^* mice. Line graph indicates the body weight of experimental group mice of increasing age. Data are expressed as mean ± SEM. Please note that the end point of each plot corresponds to the post-natal age at which all animals of each experimental group were still alive.


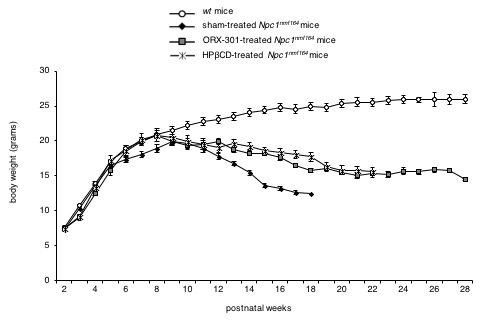


**Figure 4:** ORX-301 and HPßCD treatments started at PN14 delay the progression of body weight loss of *Npc1^nmf164^* mice at different rates. Line graph indicates the body weight of experimental group mice of increasing age. Data are expressed as mean ± SEM . See Table 6 and 8 for raw data and statistical analyses. Please note that the end point of each plot corresponds to the post-natal age at which all animals of each experimental group were alive.
